# Sinus node dysfunction after surgical atrial fibrillation ablation with concomitant mitral valve surgery: Determinants and clinical outcomes

**DOI:** 10.1371/journal.pone.0203828

**Published:** 2018-09-12

**Authors:** Darae Kim, Chi Young Shim, Geu-Ru Hong, In Jeong Cho, Seung Hyun Lee, Hyuk-Jae Chang, Sak Lee, Jong-Won Ha, Byung-Chul Chang

**Affiliations:** 1 Cardiology Division, Severance Cardiovascular Hospital, Yonsei University College of Medicine, Seoul, Republic of Korea; 2 Department of Thoracic and Cardiovascular Surgery, Yonsei University College of Medicine, Seoul, Republic of Korea; University of Milano, ITALY

## Abstract

**Objective:**

We sought to investigate determinants and prognosis of sinus node dysfunction (SND) after surgical ablation of atrial fibrillation (AF) with concomitant mitral valve (MV) surgery. A total of 202 patients who underwent surgical AF ablation with concomitant MV surgery were studied.

**Study design and setting:**

SND was defined as electrocardiographic manifestations, such as junctional bradycardia, symptomatic sick sinus syndrome, or symptomatic sinus bradycardia, 7 days after surgery. Baseline clinical and echocardiographic characteristics, rhythm outcomes [AF recurrence or permanent pacemaker (PM) implantation] at 6 and 12 months, and clinical outcomes were compared between patients without SND (n = 165) and those with SND (n = 37) after surgery.

**Results:**

Patients with SND showed a significantly larger left atrial volume index (LAVI) and a higher right ventricular systolic pressure than those without SND. In addition, there was a higher likelihood for AF recurrence and PM implantation in patients with SND than in those without SND. Although clinical outcomes did not differ between the two groups, patients with SND had a significantly longer length of hospital stay (p<0.001). In a multivariate analysis, preoperative LAVI was a structural risk factor for SND [hazard ratio (HR): 1.126 per 10 mL/m^2^; 95% confidence interval (CI): 1.0206–1.236; p = 0.001]. An LAVI cut-off value of 105 mL/m^2^ showed significant predictive power for SND [sensitivity: 62%; specificity: 64%; area under the curve (AUC): 0.678; p = 0.002].

**Conclusions:**

In conclusion, preoperative LA size was a structural risk factor for SND after surgical AF ablation during MV surgery. SND was associated with an increased risk for AF recurrence and implantation of permanent PM in patients undergoing concomitant surgical ablation of AF with MV surgery.

## Introduction

Atrial fibrillation (AF) is the most common cardiac arrhythmia among patients undergoing mitral valve (MV) surgery. The presence of AF is associated with increased risk of stroke, attenuation of reverse left atrial remodeling, aggravation of tricuspid regurgitation by annular dilatation, and increased mortality after MV surgery [1, 2]. Therefore, concomitant surgical ablation of AF during MV surgery has been widely adopted [3].

However, patients with MV disease and AF undergo structural remodeling of the left atrium (LA) as well as electrical remodeling. Structural remodeling of the LA involves increased collagen deposition, loss of myocytes, and fibrosis of atrial tissue; and, the degree of LA structural remodeling is likely to be associated with a higher susceptibility of sinus node dysfunction (SND) or AF recurrence [4].

A few studies reported the incidence of postoperative SND and pacemaker (PM) implantation after concomitant surgical ablation with MV surgery. Postoperative SND is known to be transient; however, some patients experience clinically significant permanent SND during the postoperative period and undergo permanent PM implantation [5]. There are few data regarding incidence, preoperative determinants, and clinical outcome of postoperative SND in patients undergoing concomitant surgical ablation [6, 7].

In this study, we hypothesized that preoperative LA size would predict occurrence of SND in patients undergoing MV surgery and concomitant surgical ablation of AF. Moreover, we also sought to find the clinical implications of postoperative SND regarding rhythm outcome, early surgical complications, and late clinical outcomes.

## Materials and methods

### Study population

A total of 256 patients who underwent surgical intervention for MV disease with concomitant surgical ablation of persistent or long-stranding persistent AF from 2009 to 2015 at the Severance Cardiovascular Hospital, Yonsei University College of Medicine, in Seoul, Korea, were retrospectively reviewed. According to the Heart Rhythm Society guideline, persistent AF was defined as AF lasting more than 7 days [3], and long-standing persistent AF was defined as continuous AF for more than 12 months [8]. Patients with previous permanent PM implantation (n = 45) or another concomitant congenital heart disease were excluded (n = 9). A total of 202 patients were included in the final analysis. None of the patients had evident or suspected SND before the surgery. All patients underwent echocardiography within a 1-month period before surgery and again 3 to 7 days after surgery. After surgery, for the duration of the hospital stay, all patients underwent daily 12-lead electrocardiograms (ECG) and 24-hour telemetry. Patients had regular follow-up visits at 3, 6, and 12 months after surgery and annually thereafter. At each visit, a 12-lead ECG and 24-hour Holter monitoring were performed. Regular echocardiography was conducted between 6 and 12 months after surgery and annually thereafter.

SND after MV surgery with concomitant surgical ablation of AF was defined as follows: occurrence of symptomatic sinus bradycardia with a rate less than 50 bpm, sinus arrest or sinus pauses longer than 3 seconds, symptomatic sick sinus syndrome, or a junctional rhythm when the temporary pacing in the immediate postoperative period was removed within 1 week after surgery. Patients were classified into two groups: patients without SND (n = 165, 81.7%) and those with SND (n = 37, 18.3%) after MV surgery.

All patients’ medical records were carefully reviewed by two experienced observers who were blinded to the echocardiography results. The Institutional Review Board of Yonsei University College of Medicine approved the present study.

### Echocardiography

All patients underwent comprehensive two-dimensional (2D) transthoracic echocardiography within 1 month before the surgery, after surgery at pre-discharge, and again 6 to 12 months postoperatively. Standard 2D and Doppler measurements were performed according to recommendations from the American Society of Echocardiography (ASE) [9]. The LA diameter (LAdia) was measured as an anteroposterior distance in the parasternal long-axis view, utilizing the inner edge method at the end of systole, when the LA chamber is at its greatest dimension. The LA volume was measured from apical four-chamber and two-chamber views. Maximal LA volume was measured at the end of systole using Simpson’s biplane method and was indexed to the body surface area as recommended by the ASE [9]. Left ventricle (LV) end-systolic and end-diastolic dimensions and wall thickness were measured using 2D echocardiography. The LV mass was calculated as recommended by the current guidelines and was indexed for body surface area[9]. Tricuspid regurgitation velocity was measured by continuous-wave Doppler, and right ventricular systolic pressure (RVSP) was calculated using the simplified Bernoulli equation. Regarding the estimation of pulmonary pressures, right atrial pressure was determined by inferior vena cava diameter and respiratory collapse, according to a report from the ASE [9]. Significant pulmonary hypertension of more than a moderate degree was defined as an RVSP of 50 mm Hg or greater.

### Surgical ablation of AF

Surgery was performed by two surgeons with identical surgical techniques. AF ablation was performed using an argon-based, flexible cryoablation system, SurgiFrost (Medtronic, Minneapolis, MN, USA), in all patients. The right atrial ablation included the cavotricuspid isthmus isolation lesion and a line from the isthmus lesion to the superior vena cava. The LA ablation was performed endocardially, before the MV procedure. A single box lesion for isolation of the pulmonary veins, a line from the pulmonary isolation lesion to the left atrial appendage, and another line from the pulmonary isolation lesion to the MV annulus, were posteriorly performed. Additional epicardial coronary sinus ablation was performed on the opposite side of the MV annular lesion. The duration of cryoablation was 2 minutes for the LA and right isthmus lesions and 1 minute for epicardial coronary sinus lesions and superior vena cava line. Occlusion or excision of the LA appendage was performed.

### Postoperative management

Patients who underwent MV implantation were placed on warfarin, with a target international normalized ratio (INR) of 2.5 to 3.0 after surgery, for the duration of their lives. For patients who underwent MV repair or biological MV implantation, warfarin was maintained with a target INR of 2.5 for 3 to 6 months after surgery.

### Endpoints

Rhythm outcomes included recurrence of AF and implantation of permanent PMs. Recurrence of AF was defined as any atrial tachyarrhythmia, including AF, atrial flutter, or atrial tachycardia, lasting longer than 30 seconds and detected after a blanking period of 1 month, as assessed by 24-hour Holter monitoring or 12-lead ECG at 6 and 12 months after surgery [8]. Early clinical outcomes included all-cause mortality, reoperation due to bleeding, and significant pericardial effusion requiring pericardial drainage within 1 month from the surgery. Late clinical outcomes included all-cause mortality and thromboembolic events.

### Statistical analysis

Categorical variables, expressed as percentages or frequencies, were compared between the two groups using the chi-square test or Fisher’s exact test. Continuous variables, expressed as either mean ± standard deviation or median with range, were compared between the two groups using Student’s t-test. Kaplan Meier analysis was performed to compare freedom from PM implantation between patients with and without postoperative SND. The Cox proportional hazards model was used to evaluate preoperative parameters for the prediction of postoperative SND. We constructed receiver operating characteristic (ROC) curves to determine the performance of LAVI and LAdia in predicting the development of postoperative SND. Statistical analyses were performed using SPSS statistical package version 22.0.

## Results and discussion

### Baseline characteristics

The incidence of postoperative SND in this study population was 37/202 patients (18.3%). The baseline clinical and operative characteristics of patients with or without SND are compared in Table 1. Mean age and gender distribution were similar in the two groups. Significantly more patients in the SND group had hypertension. Significantly more patients with postoperative SND underwent surgery because of severe MR (67% vs. 48%, p = 0.044). The etiology of MR according to Carpentier’s classification was similar between the two groups. Patients with postoperative SND tended to have more repairs (54% vs. 37%, p = 0.065) and fewer replacements (46% vs. 64%, p = 0.062) than those without postoperative SND, although these findings were not statistically significant. Concomitant tricuspid annuloplasty (TAP) was more frequent in the postoperative SND group than in those without postoperative SND (70% vs. 51%, p = 0.024). Duration of cardiopulmonary bypass time or aortic cross clamping time, size of valve or ring, and valve type (mechanical vs. tissue) did not differ between the two groups.

**Table 1:** Baseline characteristics.

	Without SND (n = 165)	With SND (n = 37)	p-value
***Demographic characteristics***			
Age, years	56±12	56±10	0.819
Male gender, n (%)	64 (39)	14 (38)	0.525
Body mass index, n (%)	24.5±18.2	23.6±3.6	0.774
Hypertension, n (%)	49 (30)	17 (46)	0.039
Diabetes mellitus, n (%)	20 (12)	7 (19)	0.189
Coronary artery disease, n (%)	11 (7)	2 (5)	0.572
CHA_2_DS_2_-VASc score	1.8±1.4	1.9±1.2	0.696
***Mitral valve characteristics***			
Mitral stenosis, n (%)	82 (50)	12 (32)	0.057
Moderate, n (%)	7 (4)	1(3)	0.999
Severe, n (%)	75 (45)	11(29)	0.098
Mitral regurgitation, n (%)	83 (50)	25(68)	0.057
Moderate, n (%)	4 (3)	0 (0)	0.999
Severe, n (%)	79 (48)	25 (67)	0.044
***Classification of MR***			
Carpentier type I	6 (4)	2 (5)	0.640
Carpentier type II	49 (30)	17 (46)	0.080
Carpentier type IIIa	25 (15)	4 (11)	0.350
Carpentier type IIIb	3 (2)	2 (5)	0.227
***Operation characteristics***			
Repair, n (%)	61 (37)	20 (54)	0.062
Replacement, n (%)	105 (64)	17 (46)	0.188
Concomitant AV surgery, n (%)	16 (10)	2 (5)	0.322
Concomitant TAP, n (%)	84 (51)	26 (70)	0.024
Duration of cardiopulmonary bypass, min	148 ± 69	147 ± 30	0.397
Duration of Aortic cross clamping, min	105 ± 34	110 ± 26	0.949
Size of ring	30 ± 2	29 ± 7	0.286
Size of valve	25 ± 9	25 ± 10	0.870
Valve type, n (%)			
Mechanical valve	85 (81)	14 (82)	0.999
Bioprosthetic valve	20 (19)	3 (18)	0.999

SND, sinus node dysfunction; LVEDD, left ventricular end-diastolic dimension; LVESD, left ventricular end-systolic dimension; LVEF, left ventricular ejection fraction; LV, left ventricle; LAdia, left atrial diameter; LAVI, left atrial volume index; RVSP, right ventricular systolic pressure; HTN, hypertension; AV, aortic valve; TAP, tricuspid annuloplasty

The comparisons of preoperative echocardiographic parameters between the two groups are described in Table 2. Patients with SND demonstrated a significantly larger LV mass index and LA size (LAVI and LAdia). RVSP was significantly higher in patients with postoperative SND, and more patients with postoperative p SND had pulmonary hypertension of a greater-than-moderate degree (p = 0.011) compared with patients without postoperative SND.

**Table 2:** Preoperative and postoperative structural and electrocardiographic characteristics.

	Without SND (n = 165)	With SND (n = 37)	p-value
***Echocardiographic characteristics***			
***Preoperative***			
LVEDD, mm	54.2±8.9	55.9±10.6	0.065
LVESD, mm	37.5±7.5	38.8±5.4	0.334
LVEF, %	61.1±10.3	63.5±6.8	0.201
LV mass index, g/m^2^	112±37	136±63	0.003
LAdia, mm	58±8	63±10	0.027
LAVI, mL/m^2^	101±40	132±60	<0.001
RVSP, mm Hg	41±13	46±13	0.041
Pulmonary HTN ≥ moderate, n (%)	34 (21)	14 (38)	0.011
***Postoperative (Predischarge)***			
Residual MR > mod	3 (1.9)	0 (0)	0.999
MDPG, mm Hg	4.0 ± 1.4	3.7 ± 1.2	0.167
LVEDD, mm	51.4 ± 6.8	53.3 ± 5.7	0.175
LVESD, mm	36.7 ± 6.6	37.6 ± 5.3	0.125
LVEF, %	57.1 ± 10.1	58.6 ± 9.2	0.429
LV mass index, g/m^2^	104.6 ± 27.9	121.6 ± 32.0	0.002
LAVI, mL/m^2^	72.3 ± 28.1	85.2 ± 36.4	0.002
RVSP, mm Hg	37.5 ± 11.4	41.5 ± 10.5	0.065
***Postoperative (6–12 months after surgery)***			
Residual MR > mod	3 (1.9)	0 (0)	0.999
MDPG, mm Hg	3.5 ± 1.2	3.9 ± 1.6	0.175
LVEDD, mm	49.9 ± 5.7	51.6 ± 4.7	0.186
LVESD, mm	34.6 ± 6.0	36.0 ± 4.0	0.272
LVEF, %	61.0 ± 9.3	60.0 ± 8.5	0.615
LV mass index, g/m^2^	97.4 ± 22.3	107.7 ± 25.0	0.045
LAVI, mL/m^2^	63.0 ± 22.9	69.0 ± 21.3	0.339
RVSP, mm Hg	31.3 ± 9.3	32.3 ± 12.5	0.673
**Electrical characteristics**			
***Preoperative***			
Ventricular rate, beats per minute	77±19	69±13	0.064
Atrial fibrillation, n (%)	165 (100)	37 (100)	-
***Postoperative 6 months***			
Ventricular rate, beats per minute	77±15	74 ±12	0.461
Atrial fibrillation, n (%)	26 (16)	14 (38)	0.003
Permanent PM implantation, n (%)	0 (0)	4 (11)	0.001
***Postoperative 12 months***			
Ventricular rate, beats per minute	73±11	67±12	0.062
Atrial fibrillation, n (%)	31 (19)	17 (46)	0.001
Permanent PM implantation, n (%)	0 (0)	7 (19)	<0.001
***Use of class III anti-arrhythmic drugs***			
In hospital	31(18)	5 (14)	0.311
Out of hospital (6 mo)	3 (2)	0 (0)	1.000
Out of hospital (12 mo)	3 (2)	0 (0)	1.000
***Use of class IC anti-arrhythmic drugs***			
In hospital	1 (0.6)	0 (0)	0.817
Out of hospital (6 mo)	25 (16)	6 (17)	0.803
Out of hospital (12 mo)	10 (6)	8 (23)	0.007

SND, sinus node dysfunction; PM, pacemaker

### Structural, rhythm, and clinical outcomes

The median follow-up duration in all patients was 27 ± 24 months. Echocardiography after 6 to 12 months of the surgery showed similar rates of residual MR, transvalvular gradients, and LV systolic function between the two groups (Table 2).

**Fig 1:**
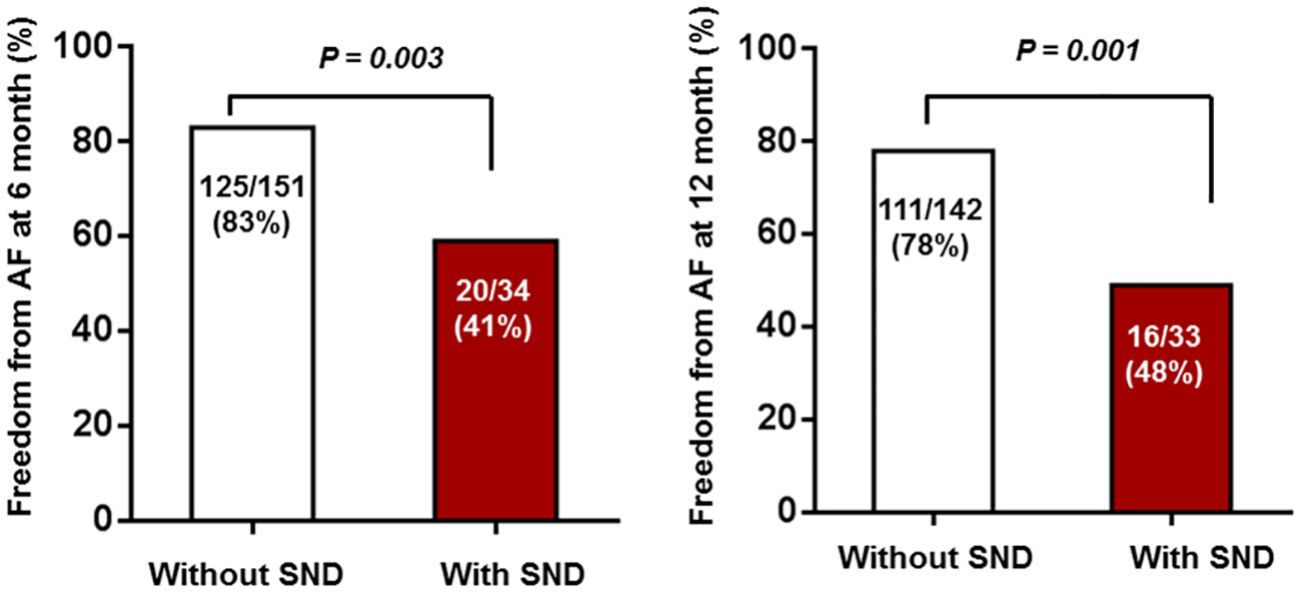
Freedom from AF at the 6-month and 12-months follow ups. Percentages of patients who were free from AF at the 6-month and 12-month follow-ups.

At the 6-month follow-up, a rhythm check-up, including ECG and 24-hr Holter monitoring, was conducted in 185/202 patients (92%) (151 patients without postoperative SND and 34 patients with postoperative SND). At the 12-month follow-up, a rhythm check-up was performed in 175/202 patients (87%) (142 patients without postoperative SND and 33 patients with postoperative SND). Freedom from AF was significantly less frequent in the SND group at the 6- and 12-month follow-ups compared with the group without SND (Fig 1, Table 2). Use of class IC anti-arrhythmic drugs was more frequent at 12 months postoperatively in patients with postoperative SND (Table 2).

**Fig 2:**
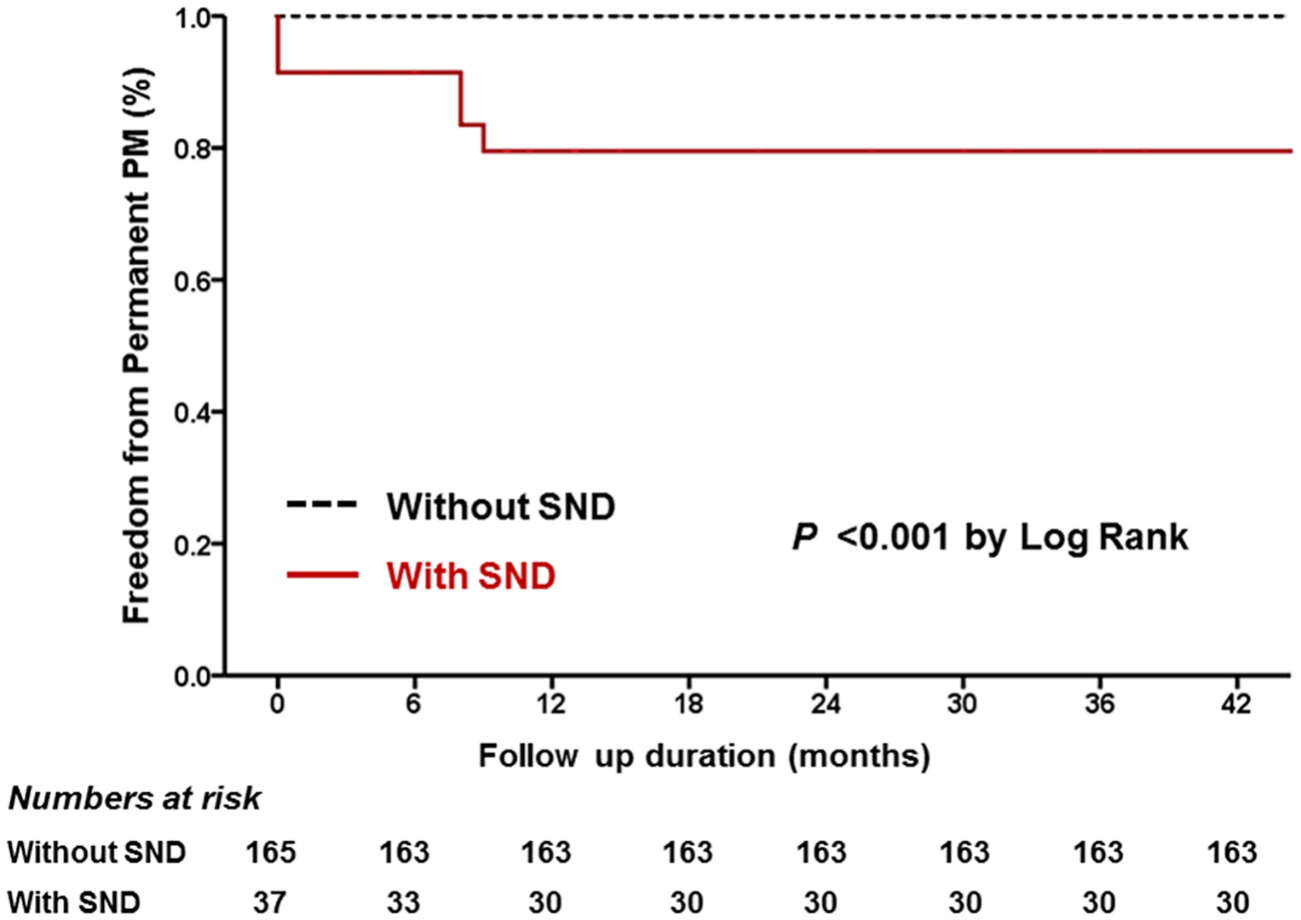
Time to event curves for permanent pacemaker implantation.

Clinical follow-ups, including those with permanent PM implantation, were conducted in all patients through regular follow-up visits and phone calls. The incidence of permanent PM implantation was significantly higher in the SND group than in the non-SND group (19% vs. 0%, respectively, at 12-month follow-up; p<0.001) (Fig 2, Table 2).

Early postoperative complications did not differ between the two groups; however, the length of hospitalization was significantly longer in the SND group than in the group without SND (p<0.001). A more frequent tendency of bleeding reoperation, wound revision, and pericardial window formation was attributed to increased hospitalization days in SND group. Also, three patents from the SND group underwent permanent PM implantation during their hospital stay after the surgery.

Late clinical outcomes, including all-cause death, thromboembolic events, redo MV surgery, and hospitalization due to heart failure, did not differ between the two groups (Table 3).

**Table 3:** Clinical outcomes.

	Without SND (n = 165)	With SND (n = 37)	p-value
***Early clinical outcomes***			
All-cause death, n (%)	2 (1)	0 (0)	0.666
Bleeding reoperation, n (%)	1 (1)	2 (5)	0.087
Wound revision, n (%)	1 (1)	2 (5)	0.087
Pericardial window formation, n (%)	7 (4)	3 (8)	0.269
Hospital stay, days	14 (11–18)	19 (13–22)	<0.001
***Late clinical outcomes***			
All-cause death, n (%)	0 (0)	1 (3)	0.183
Thromboembolic events, n (%)	10 (6)	3 (8)	0.437
Hospitalization due to heart failure, n (%)	18 (11)	3 (8)	0.425
Redo-valve surgery, n (%)	4 (2)	0 (0)	0.422

### Contributing factors for development of SND after concomitant AF ablation and MV surgery

**Fig 3:**
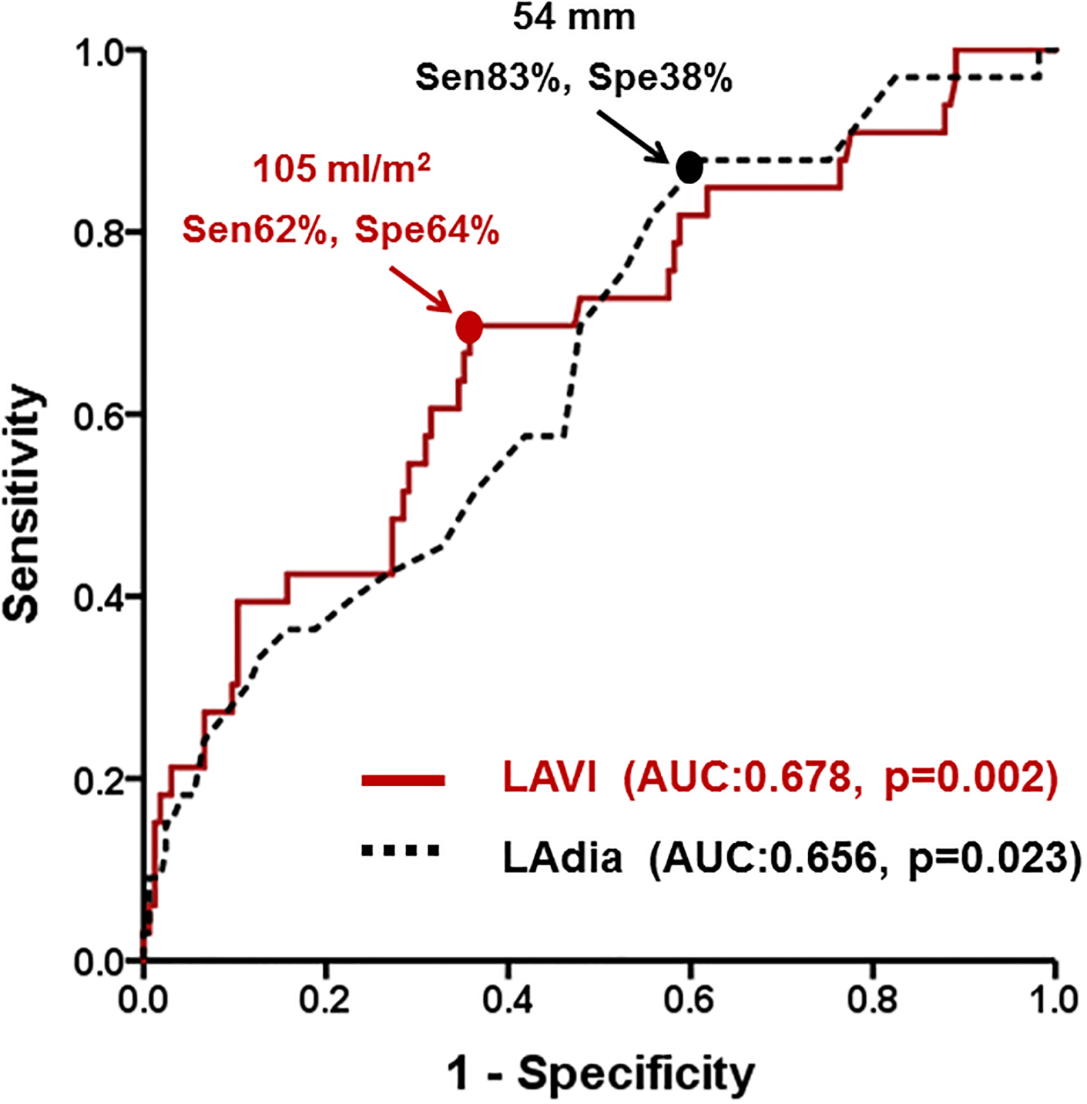
Performances of LAVI and LAdia for prediction of SND.

Table 4 shows the preoperative clinical and echocardiographic variables in association with postoperative occurrence of SND. In multivariate analysis, preoperative LAVI was found to be an independent determinant of SND (HR: 1.126 per 10 mL/m^2^; 95% CI: 1.026–1.236; p = 0.001), even after controlling for confounding factors of age, gender, indication for surgical referral (MS vs. MR), preoperative LV mass index, preoperative RVSP, and concomitant TV surgery. Both LAVI and LAdia showed satisfactory performances in predicting development of SND after MV surgery (Fig 3). A cut-off LAVI value of 105 mL/m^2^ was found to be a significant determinant for development of SND [sensitivity: 62%; specificity: 64%; area under the curve (AUC): 0.678; 95%CI: 0.568–0.769; p = 0.002], whereas a cut-off LAdia value of 54 mm was sufficient to predict SND (sensitivity: 83%; specificity: 38%; AUC: 0.656; 95%CI: 0.518–0.727; p = 0.023).

**Table 4:** Predictors of SND after surgical AF ablation in patients undergoing MV surgery.

	Hazard ratio	95% CI	p-value
Age	0.985	0.962–1.027	0.810
Male gender	1.149	0.464–2.849	0.764
Indication of MV surgery (MS vs. MR)	1.418	0.459–4.061	0.515
Preoperative LAVI, (per 10 mL/m^2^)	1.126	1.026–1.236	0.001
Preoperative LV mass index, g/m^2^	1.008	1.003–1.022	0.164
Preoperative RVSP, mm Hg	1.012	0.979–1.046	0.481
Concomitant TAP	1.664	0.663–4.174	0.278

LA, left atrium; LV, left ventricle; RVSP, right ventricular systolic pressure; TAP, tricuspid annuloplasty; AV, aortic valve

The principal findings of the present study are the following: 1) preoperative LAVI is a structural risk factor for occurrence of SND in patients who undergo surgical ablation of AF with concomitant MV surgery; 2) patients with SND after surgical ablation of AF with concomitant MV surgery more frequently developed recurrent AF and underwent permanent PM implantation; and 3) patients with SND had a longer hospitalization period, although the development of SND did not cause a significant impact on early or late clinical outcomes.

We hypothesized that there is a subset of patients who do not benefit from concomitant surgical ablation of AF when undergoing MV surgery. In our study, patients with postoperative SND showed significantly larger LA size (as defined by both LAdia and LAVI) and LVMI and higher RVSP. Significantly more patients with SND presented with pulmonary HTN of a greater-than-moderate degree and underwent concomitant TAP compared with patients with SND. Patients with postoperative SND were more likely to experience recurrent AF and were at increased risk of permanent PM implantation. In addition, patients with SND had a longer duration of hospitalization during the postoperative period than those without SND. However, neither early nor late clinical outcomes differed between the two groups.

### Clinical implication of postoperative SND after surgical ablation of AF

Since the first introduction of surgical ablation of AF, this surgical procedure has been modified to reduce the high incidence of SND. A wide range of electrographic abnormalities associated with SND, including sinus bradycardia, sinus pauses, sinoatrial blocks, and junctional rhythms, after combined surgical AF ablation and MV surgery are known to be asymptomatic and transient. Documented mechanisms of postoperative SND include injury of the nodal tissue by mechanical trauma due to atrial incisions, surgical interruption of nodal arterial supply, and inadequate atrial preservation during cardiopulmonary bypass [10–13]. There are few studies investigating clinical outcomes associated with SND after concomitant surgical AF ablation and MV surgery. SND is considered to decrease in duration, intensity, and frequency in a time-dependent manner during the first 6 months after surgery [14].

In our study, seven patients with postoperative SND underwent permanent PM implantation during a mean duration of 146 days after initial MV surgery (Table 1), whereas none of the patients without postoperative SND underwent permanent PM implantation during the follow-up period. The mean age of the seven patients was 59 ± 6 years. The finding that patients with SND needed permanent PM implantation at a younger-than-average age and after a relatively short time from surgery suggests that SND associated with AF ablation and MV surgery was likely the cause of permanent PM implantation, rather than SND that occurred as part of the aging process. Also, the prevalence of recurrent AF was significantly higher in patients with SND compared with those without SND. Together, our results suggest that the efficacy of surgical AF ablation in patients who ultimately developed SND was not as good as in patients who did not develop SND. Baseline echocardiographic parameters in the SND group showed much larger LAVI and LVMI, as well as higher RVSP compared with the non-SND group. This implies that patients who developed SND had more advanced MV disease with more arrhythmogenic atria, which are susceptible to AF recurrence. This might explain the lower efficacy of surgical AF ablation in this group of patients.

### Left atrial size as a structural risk factor of postoperative SND

The efficacy of surgical ablation of large atria is controversial. Previous studies have reported conflicting results regarding the efficacy of surgical AF ablation in patients with an enlarged atrium. Yuda et al.[15] reported restoration of sinus rhythm in 58% of patients with giant left atrium (LAdia≥ 60 mm in B mode echocardiography) at 12 months compared with 81% in patients without giant left atrium. On the other hand, Kim et al.[14] demonstrated good results of concomitant surgical ablation of giant left atrium (LAdia > 60 mm) (freedom from AF: 68.9% in the MAZE group vs. 9.6% in the no MAZE group). Various rhythm monitoring strategies, surgical experience, and different patient populations could have contributed to these heterogeneous results. However, controversial results of surgical ablation in huge atria imply that efficacy and possible complications, such as development of SND, should be taken into account.

The association between LA fibrosis and SND has been studied previously. Magnetic resonance imaging (MRI) of patients requiring PM implantation showed good correlations between the degree of LA and right atrial (RA) fibrosis[16]. Considering increasing evidences suggesting that LA size, although indirect, reflects the degree of atrial fibrosis, LA size could be used as a surrogate marker reflecting RA fibrosis [17]. The association between LAVI and SND in our results suggests that LA size may reflect functional status RA in patients with long-term AF and MV disease and susceptibility for SND. Therefore, size of LA should be taken into consideration before the surgery to reduce the occurrence of postoperative complications, such as permanent PM implantation, because the larger the LA size, the more extensive AF may exist, increasing the susceptibility of clinically significant SND.

From our results, preoperative LAVI was the only independent determinator of development of postoperative SND after adjusting for clinical and echocardiographic variables. Both LAVI and LAdia showed satisfactory performance for postoperative SND. Therefore, using left atrial size to determine high-risk patients might result in reduced occurrence of SND after concomitant surgical AF ablation and MV surgery. Patients with a large LA (LAVI >105 mL/m^2^ or LAdia > 54 mm) were found to be at a higher risk for developing SND, which in turn resulted in an increased risk for permanent PM implantation and reduced efficacy of concomitant surgical ablation of AF. In this group of patients, different strategies regarding management of AF, such as administration of anti-arrhythmic medications, cardioversion, or radiofrequency ablation, might be beneficial. Further prospective studies are needed to determine which strategies yield the best prognosis.

#### Study limitations

The main limitation of this study was that it is a retrospective study performed in a single center. All patients were diagnosed with persistent AF by serial ECGs, but identification of exact duration of AF was limited because of the retrospective nature of this study. Although we included patients without history of SND before the operation, preoperative SND was not fully assessed by electrophysiological studies. We used regular 24-hour Holter monitoring to detect recurrence of AF, which could have resulted in underestimation of the recurrence of AF, as detection rates of AF increase with long-term monitoring. Also, only 87% of patients underwent regular ECG and 24-hour Holter monitoring at the 12-month follow-up. However, data for clinical outcomes and incidence of permanent PM implantation were collected from regular follow-up clinical visits or phone calls in all patients. Therefore, despite the retrospective design of the study, we were able to conclude the prognosis of postoperative SND. Second, the SA node damage from a long vertical posterolateral cryolesion from SVC to IVC might influence the incidence of SND, and the possibility of SA node damage is less likely compared to the other techniques for AF ablation.

## Conclusions

In conclusion, enlarged LA size is a structural risk factor for SND after surgical ablation of AF with concomitant MV surgery. Moreover, the development of postoperative SND is associated with increased risk for permanent PM implantation and AF recurrence after concomitant surgical AF ablation. Therefore, preoperative LA size could provide valuable information for suitable patient selection for concomitant surgical AF ablation and might result in better patient outcomes.

## Supporting information

S1 File Supplementary Table. Clinical information of patients who underwent permanent pacemaker implantation after MV surgery with concomitant surgical ablation of AF.
(DOCX)

S2 File Data excel. (XLSX)

## Abbreviations

AF: atrial fibrillation
LA: left atrium
LAdia: left atrial diameter
LAVI: left atrial volume index
LV: left ventricle
MV: mitral valve
RA: right atrium
RVSP: right ventricular systolic pressure
SND: sinus node dysfunction
