# Supplementary material for: Sinus node dysfunction after surgical atrial fibrillation ablation with concomitant mitral valve surgery: Determinants and clinical outcomes
Source: PLoS One. 2018 Sep 12;13(9):e0203828. doi: 10.1371/journal.pone.0203828 (PMC6135507; doi:10.1371/journal.pone.0203828)
Supplement: S1 File — Clinical information of patients who underwent permanent pacemaker implantation after MV surgery with concomitant surgical ablation of AF. (DOCX) [file pone.0203828.s001.docx]

**Supplementary Table 1.**

Clinical information of patients who underwent permanent pacemaker implantation after MV surgery with concomitant surgical ablation of AF.

|  |  | **MV pathology** | **Operation** | **SND** | **Reason for PM implantation** | **Time*** |
| --- | --- | --- | --- | --- | --- | --- |
| **Patient 1** | M/63 | Severe MR  (prolapse) | MVR with TAP | + | SSS | 12 days |
| **Patient 2** | M/55 | Severe MR  (flail) | MV repair with TAP | + | SSS | 21 days |
| **Patient 3** | F/54 | Severe MS  (rheumatic) | MVR with TAP | + | SSS | 8 months |
| **Patient 4** | M/56 | Severe MS  (rheumatic) | MVR with  CABG | + | SSS | 8 months |
| **Patient 5** | F/59 | Severe MS  (rheumatic) | MVR | + | Tachy-bradycardia | 14 days |
| **Patient 6** | F/70 | Severe MR  (prolapse) | MV repair with TAP | + | Sinus pause | 9 months |
| **Patient 7** | F/56 | Severe MR  (prolapse) | MV annuloplasty  with TAP | + | SSS | 25 days |

* Time gap between surgery and implantation of permanent PM implantation

MV, mitral valve; SND, sinus node dysfunction; PM, pacemaker; MR, mitral regurgitation; MVR, mitral valve replacement; TAP, tricuspid annuloplasty; SSS, sick sinus syndrome; MS, mitral stenosis; CABG, coronary artery bypass graft
